# CRANKITE: A fast polypeptide backbone conformation sampler

**DOI:** 10.1186/1751-0473-3-12

**Published:** 2008-06-24

**Authors:** Alexei A Podtelezhnikov, David L Wild

**Affiliations:** 1 Keck Graduate Institute of Applied Life Sciences, Claremont, CA, USA; 2 Department of Physics, Michigan Technological University, Houghton, MI, USA; 3 Systems Biology Centre, University of Warwick, Coventry, UK

## Abstract

**Background:**

CRANKITE is a suite of programs for simulating backbone conformations of polypeptides and proteins. The core of the suite is an efficient Metropolis Monte Carlo sampler of backbone conformations in continuous three-dimensional space in atomic details.

**Methods:**

In contrast to other programs relying on local Metropolis moves in the space of dihedral angles, our sampler utilizes local crankshaft rotations of rigid peptide bonds in Cartesian space.

**Results:**

The sampler allows fast simulation and analysis of secondary structure formation and conformational changes for proteins of average length.

## Background

One important challenge of structural protein modeling is an efficient sampling technique for rapid search through the enormous conformational space [1]. Monte Carlo (MC) simulations, along with molecular dynamics, are among the most commonly used methods of sampling conformational space [2]. Because peptide bonds are rigid and flat, MC simulations are often performed in the space of dihedral ϕ-ψ angles, which reduces the number of degrees of freedom and speeds up simulations [3]. In addition, the conformations of just a few amino acids are perturbed locally on each step, leaving the rest of the chain intact, which increases the acceptance probability of the attempted moves and the efficiency of Metropolis MC procedure [4].

As an alternative to simulations in dihedral space, we modeled rigid peptide bonds explicitly and used local crankshaft rotations in Cartesian coordinates to displace them. An important feature of our model is the elasticity of the alpha carbon valence geometry. With flexible alpha carbon valence angles, it becomes possible to use crankshaft moves inspired by earlier Metropolis MC studies of large-scale DNA properties [5,6]. Our polypeptide model features all-atom representations of the polypeptide backbone as well as beta-carbon atoms. Other side-chain atoms are omitted from consideration. We also draw on parallel tempering (replica exchange) [7] to speed up equilibration of the system when necessary. A detailed description of the model is provided in our earlier publication [8].

## Methods

**Figure 1:**
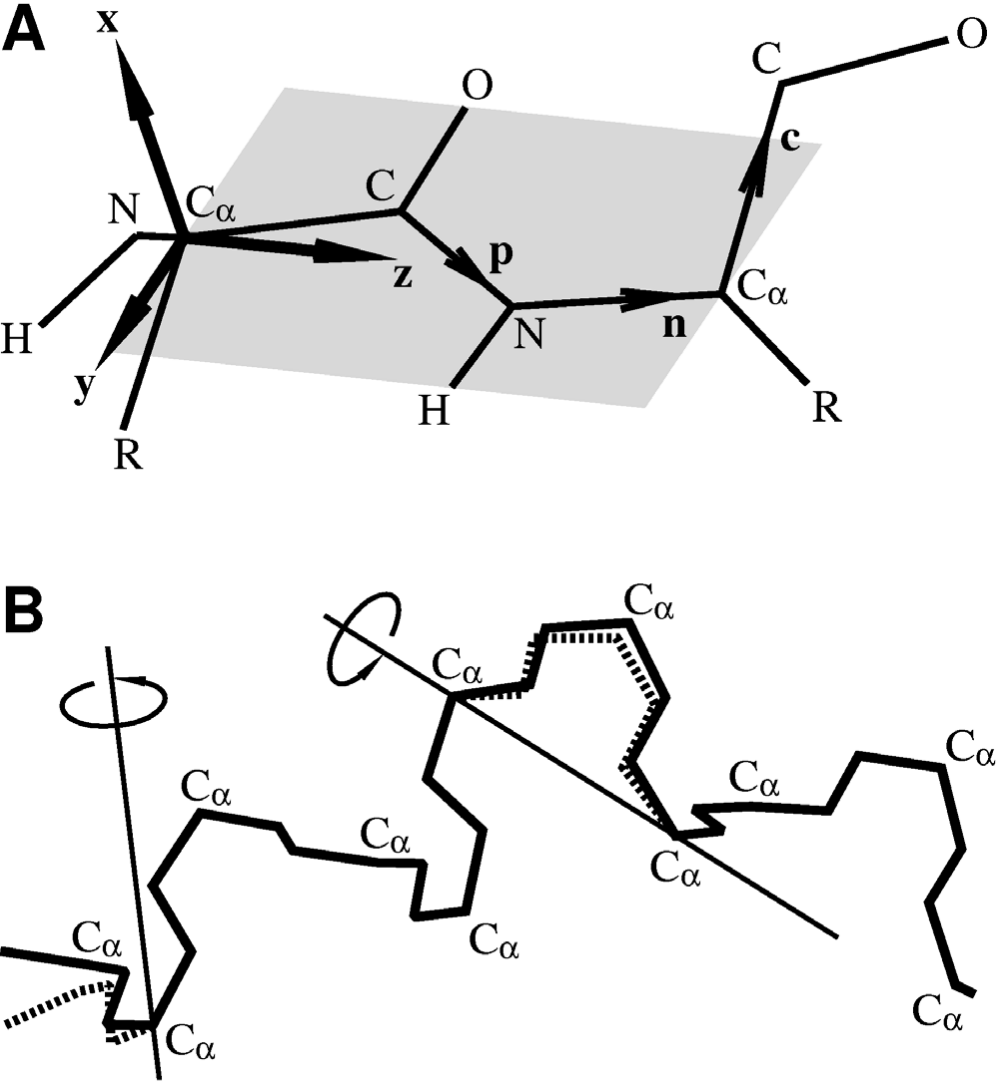
(**A**)** Polypeptide model**. The orientations of perfectly planar and rigid peptide bonds are given by the orthonormal triplets (**x**, **y**, **z**), with **z **pointing along the C_α_-C_α _direction. Other peptide bond atoms lie in the plane *yz*. The position of the side-chain atoms R is specified by the vectors **n **and **c**. (**B**) Local Metropolis moves. Two types of moves are used in this work: a crankshaft rotation around the line connecting 2 C_α _atoms in the middle of the chain, and a random rotation at the termini around a random axis passing through the C_α _atom. From Podtelezhnikov and Wild (2005) [8].

In our model, the primary descriptors of the polypeptide chain conformation are the orientations of the peptide bonds in the laboratory frame (Fig. 1A). For a chain of *N *amino acids the orientations of the peptide bonds are specified by the orthonormal triplets, (**x**_*i*_, **y**_*i*_, **z**_*i*_), *i *= 0, ..., *N*. The vector **z**_*i *_points in the direction from ${\text{C}}_{\alpha }^{i}$ (alpha carbon of amino acid *i*) to ${\text{C}}_{\alpha }^{i+1}$. The distance between the alpha carbons is fixed and equal to 3.8 Å for a peptide bond in trans conformation. The peptide bond atoms lie on the *yz *planes, with backbone geometry corresponding to the classical average bond lengths and angles [9]. The direction of the C_α_-C_β _bond corresponds to tetrahedral valence geometry and chirality of L-amino acids, see Fig. 1A. The C_α_-C_β _bond length is equal to *b *= 1.531 Å [9].

To obtain the canonical ensemble of polypeptide conformations, we developed a novel Metropolis Monte Carlo procedure. Each new chain conformation was generated from the previous one by applying a move to a randomly chosen chain segment containing one or two adjacent peptide bonds. Each move was local and the conformation of the rest of the chain was not altered. Local moves are extremely important in achieving an efficient sampling of the polypeptide conformational space [4]. Two types of moves used in our procedure were random rotations by a small uniformly distributed angle. The move of the first type was a crankshaft rotation of the peptide bonds between randomly picked *i *and *j*, with the line connecting ${\text{C}}_{\alpha }^{i}$ and ${\text{C}}_{\alpha }^{j}$ as the rotation axis (Fig. 1B). We applied the same rotation to the triplets (**x**_*i*_, **y**_*i*_, **z**_*i*_) through (**x**_*j*-1_, **y**_*j*-1_, **z**_*j*-1_) and, then calculated the new positions of the backbone atoms. As a result, some or all atoms of the amino acids *i *though *j *were displaced while the peptide bonds remained planar. The move of the second type involved a rigid-body rotation of one or two peptide bonds at one of the chain termini picked randomly with rotation axis passing through the alpha carbon in a random direction (Fig. 1B). In the current implementation, up to 4 peptide bonds are rotated on each step. It is important that in this scheme any rotation and its inverse are picked with equal probability.

This Metropolis procedure in Cartesian space would be impossible without making the alpha carbon valence geometry flexible in our model. This is an extra degree of freedom that is usually fixed in dihedral space simulations. The dihedral space simulations, however, require complex computations of dihedral angle moves (so called "re-bridging" or "loop-closure") so that the structure only perturbed locally [10,11]. In addition, sampling in dihedral space require the calculation of a Jacobian correction to satisfy the microscopic reversibility principle and achieve unbiased sampling of dihedral angles [12,13]. The crankshaft rotations in Cartesian space are, on the other hand, trivially reversible and proposed with equal probabilities. Therefore, the Metropolis acceptance criterion did not require the Jacobian correction. In our previous work, this was further validated by demonstrating unbiased sampling of ϕ-ψ angles using crankshaft rotations [8]. We believe that the simplicity of our Metropolis MC procedure is well worth adding of an extra degree of freedom to our model.

Crankshaft rotations have been used in polymer simulations for decades [14]. Recently, Betancourt [15] wrongly suggested that crankshaft rotations require a correction in the Metropolis acceptance rate to achieve proper sampling of dihedral and bond angles. This paper erroneously treated dihedral and bond angles as multi-dimensional Cartesian space rather than regular polar coordinates. In fact, no correction is necessary and crankshaft rotations achieve proper sampling automatically.

We implemented simplified interaction potentials between backbone atoms and side-chains. Van der Waals interactions were mimicked by hard-sphere repulsion between atoms. Hydrogen bonding interactions were modeled by square-well potentials. To simulate interactions between side-chains, we specified Go-type potentials [16] between the C_β _atoms that were explicitly implemented in our model.

Sampling results are produced in a PDB format ready to be analyzed and visualized by a variety of available tools. The program also allows monitoring contacts between residues, hydrogen bonding patterns, radius of gyration, etc. during simulations. This suite also contains helper programs to create initial conformations by specifying arbitrary dihedral angles and to calculate contact maps and dihedral angles in the simulated trajectory. CRANKITE is implemented in ANSI C. It has been tested to compile with GCC, Sun Studio, and MIPSPro compilers and run under Linux, Solaris, and IRIX. Parallel tempering for the conformation sampler has been implemented using MPI 1.2 and tested on a Sun NETRA X1 Cluster Grid.

## Usage

The package provides a Makefile recognized by GNU *make *(or *gmake*), which builds the suite. Most programs in the suite are controlled through various options given on the command line. Invalid options (or the valid option "-h") trigger the output of usage notes. The following programs are provided in the suite from most to least important.

### peptide

The Metropolis polypeptide backbone conformation sampler. A very short simulation can be run by using a command like

./peptide -r 1000x25 -t 201 d1ctf__.pdb

This will read the initial conformation from file d1ctf__.pdb, perform 25,000 Metropolis moves, while recording a contact map and a snapshot conformation every 1000 steps. The output will have 25 contact maps and 25 snapshots. To get help with more options, use "./peptide -h".

### peptmpi

The same as peptide, but compiled for parallel-tempered simulations under MPI.

### rama

Calculates backbone dihedrals for polypeptides in a PDB file. The valence geometry of each CA atom is characterized in terms of N-CA-C valence angle τ and normalized solid angle formed by three bonds with heavy atoms. The solid angle is positive for L-amino acids and negative for D-amino acids. Side-chain dihedral angles χ_1 _are also reported. Other calculated characteristics of local polypeptide conformation include CA-CA and CB-CB distances between adjacent residues, pseudo-dihedral angles CB-CA-CA-CB, etc.

### lipa

Converts dihedrals to a 3D-structure in PDB format. This program may be used to create an arbitrary initial conformation for *peptide*. Each line of the input should start from a 1-letter residue id and dihedral angles ϕ-ψ separated by spaces, e.g. "Q -57 -47". Optionally, valence angle τ and cis/trans angle ω can be given in the fourth and fifth positions. This program is an approximate inverse of *rama *and one can pipe *rama *output through *lipa *to recreate the original structure. This restoration is not ideal, because lipa only uses values of ϕ, ψ, and τ.

### coma

Calculates a contact map for a protein structure in a PDB file. Contacts are defined by a cut-off distance between CA or CB atoms. Some of this functionality is also integrated in *peptide*. The program can also produce a distance map.

### befa

Produces a mean structure with B-factors for the multiple models in an NMR-style PDB file. Calculation of B-factors for a simulation trajectory is reliable only if the trajectory is very short (less than 100 steps per residue).

### stats

Basic statistical pipe tool to calculate means, standard deviations, statistical inefficiencies and extreme values for a sequence of numbers separated by whitespace.

### merg.sh

Merges and sorts output files that are produced during a Sun MPI parallel-tempered run using "mprun -B". The files to be merged should be listed on the command line. The script and the *merg *executable are useful only on Sun clusters.

### cdlearn.sh

An example script to set up Contrastive Divergence learning (see below) from a dataset of PDB structures. The script uses 2 parameters: the number of available cluster nodes and a file with the list of PDB id's. This script distributes tasks over cluster nodes using PBS (Portable Batch System). Each node receives a PBS job *diverge.pbs*.

### viewer.sh

PDB viewer produces a stereo pair in a printable PostScript format. It uses a PDB file name as a command line parameter. PDB files are parsed by the *oops *executable that is called from the script.

By default, all the programs read data from the standard input (stdin) and write to the standard output (stdout), so that one can use them in a pipeline. If a file name is given on the command line, the input is redirected from this file. The option "-o filename" allows one to redirect the output to a file. This is an example of a pipeline that involves most programs in the suite

./rama d1ctf__.pdb | ./lipa | ./peptide -r 500x10 | ./befa | ./coma

Here, *rama *will calculate dihedral angles for d1ctf__ and pass them to *lipa *to recreate a 3d structure, which is passed to *peptide *for a short simulation producing 10 snapshots, then *befa *averages them, and finally *coma *produces a contact map for the average structure.

We implemented an alternative way of setting initial conformation for *peptide*. One can enter a sequence of residues directly on the command line, e.g.,

./peptide -r 4096x200 ABCDEFGHabcdefgh

This will run simulations on the 16-residue polypeptide, starting from the first 8 residues in alpha-helical state and the last 8 residues in beta-strand conformation.

The default force-field used by peptide includes hard-core van der Waals repulsions and square-well interpeptide hydrogen bonding. One can also specify Go-type interactions between side-chains by providing a regularized contact map. For example,

./peptide -r 4096x200 -p _B=beta-16.in ABCDEFGHABCDEFGH

where the file beta-16.in (provided in the package) contains the regularized contact map corresponding to 16-residue beta-hairpin. The above example starts simulations from alpha-helical conformation and turns it into a beta-hairpin in less than 15 seconds on a 3.2 GHz Pentium D system.

The *peptide*'s option "-p" is perhaps the most powerful option, which allows one to control many parameters of the program, including force-field constants. This option is intended to be customizable with flexible format. To learn more about this option in the current version, we refer the reader to the source code.

## Application internals

The peptide backbone sampler and most of helper programs are implemented in ANSI C. A Makefile to be used with GNU make was tested with GNU, Sun Studio, and MIPS Pro C compilers. Below we describe some details of the Metropolis sampler implementation.

### main.c

Mostly contains the main simulation loop and its setup from command line parameters. Other tasks implemented in this file include temperature setup and updates for parallel-tempering or annealing simulations.

### metropolis.c

Contains the implementation of the Metropolis procedure including the initial conformation setup and updates by Metropolis moves. The major data structures are declared, allocated and initialized in this file.

triplet *xaa, *xaat;

are arrays of orthonormal triplets describing current and trial orientations of planar peptide bonds.

struct AA *aa, *aat;

are arrays of structures with current and trial individual amino acid descriptions and atomic coordinates.

double *erg, *ergt;

are arrays with current and trial pairwise interaction energies between amino acids.

int cranckshaft ();

implements crankshaft rotations.

### peptide.c

Implements the polypeptide model with rigid planar peptide bonds with fixed bond lengths and angles. It also contains routines for input and output of coordinates in PDB format.

void carbonate_f (); or void carbonate_b;

places C_α _based on previous or next C_α _and peptide bond orientation.

void acidate ();

rebuilds amino acid atoms based on alpha carbon position and orientations of peptide bonds around it.

void amidorient ();

determines orientation of peptide bond between two amino acids.

void pdbrecord ();

outputs coordinates in PDB format.

### probe.c

Implements tests and measurements that are to be performed during simulations (e.g., radius of gyration, current total energy, or dihedral angles).

### energy.c

Contains the force field implementation. It implements interactions between amino acids by specifying interactions between their individual atoms that are included in the model. It also contains tests that depend on the force-field implementation.

double energy1 ();

returns energy of interactions inside a particular amino acid.

double energy2 ();

returns energy of interactions between two amino acids.

### aadict.c

Implements conversions between standard 1- and 3-letter amino acid abbreviations.

### rotation.c

Implements manipulation of orientation triplets (e.g., rotation matrices, matrix-triplet and matrix-vector multiplication, or Euler angles determination).

### vector.c

Implements vector routines (e.g., dot product, cross product, or dihedral angles).

## Benchmarking

It is not always easy to benchmark software that is designed with a particular purpose in mind. Biochemical software is not an exception. We were able to compare CRANKITE sampling performance with CNS, which is widely used in a variety of biochemical simulations ranging from structure refinement to torsion dynamics [17]. We compared the time it takes for both suites to bring two β-strands together forming an anti-parallel β-hairpin. In the initial conformation β-strands are separated by a 45° angle. The peptide contained 16 alanine residues. We used a Go-type potential between C_β _atoms of lateral β-hairpin neighbors to bias the sampling towards the formation of the β-hairpin. In CNS the biasing was achieved by means of soft pseudo-bonds between the C_β _atoms. The force constants of the biasing potential were identical in the CRANKITE and CNS simulations. In the CNS molecular dynamics simulations, the conformations resembling a β-hairpin started to appear after about 0.4 ns. With the aggressive choice of time step of 0.004 ps, the simulations took about 1 min on a 3.2 GHz Pentium D computer. In the CRANKITE Metropolis Monte Carlo simulations, β-hairpin conformations started to appear after about 100,000 steps. It took less than 2 seconds to complete this simulation run on the same system. Therefore, in this test, CRANKITE seems to be 30 times faster than CNS.

**Figure 2:**
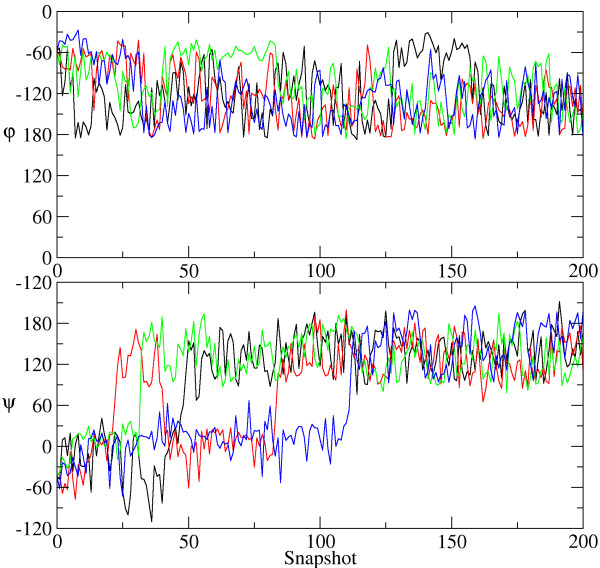
**Dihedral angle evolution**. In these simulations a 16-mer polypeptide underwent conversion for an α-helix to a β-sheet conformation. Changes in dihedral angles for residues 3, 4, 5, 6 are shown in black, red, blue, and green respectively. Snapshots were collected every 4096 steps.

CRANKITE was designed to perform quick conversions between secondary structure elements. It converts a 16-residue α-helix into a β-hairpin in less than 1 million Metropolis moves (see Usage example above). Fig. 2 shows examples of dihedral angle evolution during these simulations. In the figure, after starting simulations with the dihedral angles corresponding to an α-helix, the residues 3, 4, 5, and 6 have adopted the angles corresponding to a β-sheet. These simulations took less than 15 seconds to complete. We were not able to complete this conversion using CNS. Although CNS is a great program for many biochemical tasks, CRANKITE excels in sampling secondary structure conversions.

## Conclusion

Using our efficient implementation of Metropolis Monte Carlo procedure for polypeptides, we were able to perform exhaustive sampling of the polyalanine conformations [8]. We have also applied this suite to study properties of interpeptide hydrogen bonding relying on a modern machine learning technique known as Contrastive Divergence [18]. This suite has also been used to simulate proteins of moderate length. We have also investigated the reconstruction of protein backbone conformations and secondary structure elements in atomic detail based on a prior prediction of the protein contact map and secondary structure (Podtelezhnikov and Wild, submitted). Our software demonstrates remarkable efficiency and reliability in performing these computer experiments and has the flexibility to be applied to many other biological problems. In releasing our software under the GNU General Public License we hope that others will contribute to its continuing evolution.

## Availability

The suite is available under the terms of GNU General Public License from .

## Competing interests

The authors declare that they have no competing interests.

## Authors' contributions

Both authors equally contributed to this work including conception and design of the software. AP implemented the software.
